# International SF-36 reference values in patients with ischemic heart disease

**DOI:** 10.1007/s11136-016-1316-4

**Published:** 2016-06-18

**Authors:** Alexandra Huber, Neil Oldridge, Stefan Höfer

**Affiliations:** 1 Department of Medical Psychology, Medical University of Innsbruck, Speckbacherstraße 23/III, 6020 Innsbruck, Austria; 2 Institute of Psychology, Leopold Franzens University Innsbruck, Innrain 52f, Bruno-Sander-Haus, 6020 Innsbruck, Austria; 3 College of Health Sciences, University of Wisconsin-Milwaukee, 6975 N. Elm Tree Road, Glendale, WI 53217 USA

**Keywords:** SF-36, Reference data, Ischemic heart disease, Angina, Myocardial infarction, Heart failure

## Abstract

**Purpose:**

International reference data for the SF-36 health survey (version 1) are presented based on a sample of 5508 adult patients with ischemic heart disease.

**Methods:**

Patients with angina, myocardial infarction and ischemic heart failure completed the SF-36. Data were analyzed by diagnosis, gender, age, region and country within region and presented as mean ± standard deviation (SD), minimum, maximum, 25th, 50th and 75th percentile of the physical (PCS) and mental component summary (MCS) measures.

**Results:**

Mean PCS scores were reported as being more than one SD below the normal range (standardized mean of 50 ± 10) by more than half of the patient subgroups (59 %) with all of the mean MCS scores falling within the normal range. Patients with angina and patients with ischemic heart failure reported the poorest mean PCS scores with both diagnoses reporting scores more than one SD below the standardized mean. Females, older patients (especially >70 years) and patients from Eastern Europe reported significantly worse mean PCS scores than male, younger and non-Eastern European patients. The cardiac diagnosis had no effect on the mean MCS scores; however, females, younger patients (especially <51 years) and patients from Eastern Europe reported significantly worse mean MCS scores than male, older and non-Eastern European patients.

**Conclusions:**

These international reference SF-36 values for patients with IHD are useful for clinicians, researchers and health-policy makers when developing improved health services.

**Electronic supplementary material:**

The online version of this article (doi:10.1007/s11136-016-1316-4) contains supplementary material, which is available to authorized users.

## Introduction

Health-related quality of life (HRQL), as an integral aspect of subjective patient-reported health status, has become an increasingly important health care outcome measure, especially in patients with chronic diseases, for example, cardiovascular disease [1–3]. In 2012, cardiovascular diseases were the number one cause of death globally with about 17.5 million people dying of cardiovascular diseases, or 31 % of all global deaths; of these deaths, approximately 7.4 million (42 %) were due to ischemic heart disease (IHD) [4]. Patient-reported health status, including HRQL, is predictive of mortality, cardiovascular events, hospitalization and costs of care in patients with cardiovascular disease; despite this, instruments to assess patient-reported health status are underused in clinical practice [1, 2].

Attributes and criteria for HRQL instruments are important as quality indicators. Key attributes of HRQL instruments include the conceptual and measurement model, reliability, validity, language adaptations and interpretability [5]. The key to the interpretation of HRQL is having reference data. Without such data, it is difficult for the user to assess the meaning of the scores because benchmark values are missing. For example, reference data allow a determination of whether group or individual HRQL scores and standard deviations are below, similar to, or above those of a reference group thus placing them into a context; furthermore, comparing percentiles and minimum/maximum values in a study sample can provide useful information on the distribution of HRQL scores [6]. A number of instruments have been developed to quantify HRQL, and some HRQL manuals offer population norms and distributions relating to gender, age or disease [7, 8] while various studies have provided within-country [9–11] and between-country [12, 13] HRQL comparative data. The Short Form-36 health survey (SF-36) [8, 12, 13] is arguably one of the most widely used generic health-related quality of life measures in the general population and also in patients with IHD [12–19].

The International Quality of Life Assessment (IQOLA) Project [12, 13] was a comprehensive project to translate, adapt and validate the SF-36 internationally in patients with chronic disease. Patients with congestive heart failure reported the second lowest SF-36 physical health, while the “effect of ischemic heart disease on a number of physical health scales was noteworthy.” The EuroAspire III study [20] used the SF-12 in patients with IHD, where lower HRQL estimates were found in women, older patients and patients from Eastern European countries. Soto et al. [15] also reported lower physical health scores for females and older patients and moreover for patients with myocardial infarction (MI) compared to patients with angina. A similar distribution was found in a study by Alphin et al. [16] where patients with heart failure reported the lowest physical health, followed by patients with MI and then angina. The impact of a chronic condition on mental health was always lower than physical health. Meta-analyses or systematic reviews showed that the SF-36 also correlates with disease-specific questionnaires like for heart failure [17] and is a predictor of health status [18] confirming its broad area of application [19].

Despite the widespread use of the SF-36 in different populations, to our knowledge, no international IHD reference data in patients with angina, MI or ischemic heart failure are available. As a result, international comparisons (especially including Eastern European countries) with data acquired on the basis of either one defined study protocol or studies by independent researchers are unavailable. Therefore, the aim of this report is to present international reference data for the SF-36 (including sub-analyses on the effect of, e.g., diagnosis, gender and age) based on a sample of 5508 adult patients with IHD and a diagnosis of angina, MI or ischemic heart failure living in one of 22 countries and speaking one of 15 languages.

## Methods

### Sample

The data analyzed in this study were generated in the HeartQoL Project where a new HRQL disease-specific questionnaire for patients with IHD—the HeartQoL questionnaire—was developed and validated [21, 22]. The HeartQoL Project is an international HRQL survey conducted between 2002 and 2011, including 6384 patients with documented angina, MI or ischemic heart failure living in five regions (Eastern, Northern, Southern and Western European regions and an English-speaking region) with a total of 22 countries where 15 languages are spoken: Danish, Dutch, English (Australia, Canada, Ireland, UK, USA), French, Flemish, German (Austria, Germany, Switzerland), Hungarian, Italian, Norwegian, Polish, Portuguese, Russian, Spanish (Cuba, Spain), Swedish and Ukrainian. Each of the sites (*N* = 67) received local Ethics Committee or Institutional Review Board approval. Following specific protocol directions (e.g., guidelines were specified in each language for identifying crucial disease symptoms, universal inclusion criteria, workshops on standardized surveying), participating physician investigators at a total of 67 sites (hospital cardiology clinics and cardiac rehabilitation programs) identified eligible patients when seen at their clinic visit; the nature and purpose of the study was then explained, and, with written informed consent, eligible patients were enrolled in the study.

#### Eligibility criteria

All HeartQoL Project patients had to be at least 18 years old, were not currently substance abusers, did not have a serious psychiatric disorder, were considered by the referring physician to be able to complete a self-administered battery of HRQL instruments in the particular language and had not been hospitalized during the past 6 weeks [21, 22]. Patient physicians reported the primary diagnosis of angina, MI or ischemic heart failure according the following criteria:

1. Currently treated for angina (typical chest pain, Canadian Cardiovascular Society (CCS) class II, III or IV) with an objective measure of IHD (exercise testing, echocardiogram, nuclear imaging or angiography); or
2. Experienced a documented MI between 1 and 6 months previously, including chest discomfort, electrocardiogram changes indicative of MI and positive creatine kinase or troponin rise; or
3. Currently treated for ischemic heart failure (New York Heart Association (NYHA) class II, III, or IV) with evidence of left ventricular dysfunction (ejection fraction <40 % by invasive or noninvasive testing) and IHD (previous MI, exercise testing, echocardiogram, nuclear imaging or angiography). Other underlying heart failure diagnoses were excluded.

### SF-36 health survey (version 1)

The SF-36 [8] consists of 36 items, each scored in one of eight scales (Physical Functioning, Role-Physical, Bodily Pain, General Health, Vitality, Social Functioning, Role-Emotional, Mental Health) which then form two distinct higher-ordered clusters—the physical component summary (PCS) and mental component summary (MCS) measure. Data from the eight scales are presented as raw values (0–100). PCS/MCS data are presented as T-scores with a mean (M) of 50 ± 10 standard deviation (SD), with higher scores indicating better HRQL. The instrument meets required psychometric standards [5] and is one of the most widely used generic HRQL measures in patients with IHD [12–19]. Reference data on the eight scales and the higher-ordered PCS/MCS measures are available [7, 8]. The first SF-36 question “In general, would you say your health is…excellent/very good/good/fair/poor?” was used to characterize the current health status of the sample (Table 1).

### Statistical analyses

Data are presented as M ± SD, lowest and highest SF-36 scores, medians and 25th and 75th percentiles including Cronbach’s Alpha α as follows; (a) socio-demographic characteristics (age, gender, education, family status); (b) clinical characteristics (angina, MI or ischemic heart failure including the SF-36 question on self-reported health, disease severity, risk factors); and (c) geographic regions. Sites were located in Eastern Europe (EE)—Hungary, Poland, Russia, Ukraine (*N* = 4 sites); English-speaking countries (ES)—Australia, Canada, Ireland/UK, USA (*N* = 19 sites); Scandinavia (Sc)—Denmark, Norway, Sweden (*N* = 11 sites); Southern Europe (SE)—Italy, Spain, Portugal (*N* = 10); Cuba (*N* = 1); and Western Europe (WE)—Austria/Germany/Switzerland, Belgium, France, Netherlands (*N* = 22). We combined German language data from Austria, Germany and Switzerland and European English language data from Ireland and UK. Cuban and Spanish patients were originally pooled in the parent HeartQoL Project to a “Spanish-speaking group” for maximizing variance but, within this analysis, patients coming from Cuba were examined separately due to economic and cultural differences. Means are provided for the PCS and MCS measures and raw values for the eight SF-36 scales. SPSS 21 was used for all statistical analyses (descriptive statistics; crosstabs; independent-samples *t* tests, uni- and multivariate analyses of variance (ANOVA; MANOVA) with Bonferroni correction (BC) investigating group differences; *z* tests for population proportions) and effect sizes (partial eta-squared (*η*_*p*_^2^) or Cohen’s d are reported. Incomplete datasets (e.g., missing values on age, gender or diagnosis, incomplete SF-36 data) and SF-36 outliers with standardized z-scores in excess of 3.29 [23] were excluded from the total dataset (description within the limitation section), leading to a cohort of 5508 patients.

## Results

### Socio-demographic and clinical characteristics (Table 1)

In this analysis, there were 1836 patients (33.3 %) with documented angina, 2086 (37.9 %) with a documented MI and 1586 (28.8 %) with documented ischemic heart failure. Socio-demographic and clinical characteristics are detailed in Table 1. Based on the first SF-36 question “In general, would you say your health is…?”, only 1.8 % of the patients rated their health as “excellent”, 47.3 % rated their health as being either “very good” or “good”, 41.5 % rated their health as “fair” and 9.4 % rated their health as “poor”. There were 1160 patients from EE (21.1 %), 1231 from ES (22.3 %), 856 from Sc (15.5 %), 793 from SE (14.4 %), 1309 from WE (23.8 %) and 159 from Cuba (2.9 %).

**Table 1:** Socio-demographic characteristics including diagnosis, age, gender, family status, education, self-reported health, region, risk factors and disease severity (data missing if sample sizes do not equal *N* or 100 % for each group)

	Analyzed cohort		Angina		MI		Heart failure	
	*N*	%	*N*	%	*N*	%	*N*	%
	5508	100.0	1836	33.3	2086	37.9	1586	28.8
**Gender**								
Female	1312	23.8	494	26.9	480	23.0	338	21.3
Male	4196	76.2	1342	73.1	1606	77.0	1248	78.7
**Age (years ± SD)**	62.0 ± 11.2		62.6 ± 10.1		59.2 ± 11.2		64.8 ± 11.5	
**Age category**								
<51	866	15.7	222	12.1	465	22.3	179	11.3
51–60	1656	30.1	579	31.5	705	33.8	372	23.5
61–70	1676	30.4	608	33.1	562	26.9	506	31.9
>70	1310	23.8	427	23.3	354	17.0	529	33.4
**Family status**								
Single	632	11.5	194	10.6	243	11.6	195	12.3
Married	4145	75.3	1395	74.4	1570	75.3	1180	74.4
Other	667	12.1	216	11.8	254	12.2	197	12.4
**Education**								
<High school	1947	35.3	674	36.7	659	31.6	614	38.7
High school	1708	31.0	587	32.0	654	31.4	467	29.4
>High school	1674	30.4	502	27.3	717	34.4	455	28.7
**Self-reported health**								
Excellent	97	1.8	24	1.3	56	2.7	17	1.1
Very good	562	10.2	162	8.9	308	14.8	92	5.8
Good	2034	37.1	627	34.3	914	44.0	493	31.2
Fair	2276	41.5	818	44.7	689	33.2	769	48.7
Poor	518	9.4	199	10.9	111	5.3	208	13.2
**Region**								
Eastern Europe	1160	21.1	398	21.7	401	19.2	361	22.8
English speaking	1231	22.3	442	24.1	437	20.9	352	22.2
Scandinavia	856	15.5	291	15.8	316	15.1	249	15.7
Southern Europe	793	14.4	262	14.3	308	14.8	223	14.1
*Cuba*	159	2.9	57	3.1	74	3.5	28	1.8
Western Europe	1309	23.8	386	21.0	550	26.4	373	23.5
**Risk factors**								
Hypertension^a^	3066	55.7	1184	64.5	1046	50.1	836	52.7
Diabetes^a^	1116	20.3	397	21.6	313	15.0	406	25.6
Hypercholesterol^a^	3305	60.0	1234	67.2	1212	58.1	859	54.2
Smoking	818	14.9	241	13.1	346	16.6	231	14.6
BMI (mean ± SD)	27.4 ± 5.0		27.9 ± 4.9		26.9 ± 4.7		27.3 ± 5.1	
Physical inactivity^b^	3693	68.0	1229	67.9	1320	64.0	1144	73.3
**Disease severity**								
CCS II			1235	67.3				
CCS III + IV			543	29.6				
NYHA II							912	57.5
NYHA III + IV							632	39.8

*BMI* body mass index, *CCS* Canadian Cardiovascular Society class, *MI* myocardial infarction, *N* number of patients, *NYHA* New York Heart Association Class, *SD* standard deviation

^a^As told by her/his physician

^b^Active on <3 occasions per week

### SF-36 component summary measures: diagnosis, gender and age (Table 2)

All M ± SD, minimum, maximum, 25th, 50th and 75th percentile PCS/MCS scores and Cronbach’s Alpha for diagnosis, gender and age are given in Table 2.

**Table 2:** Physical component summary (PCS) and mental component summary (MCS) mean scores, standard deviations and Cronbach’s Alpha by diagnosis, gender and age; grouped by a combination of gender-diagnosis, age-diagnosis and age-gender-diagnosis (mean PCS or MCS scores >1 standard deviation below the standardized mean of 50 are bold)

	Mean | standard deviation | α		Min | max score		25th | 50th | 75th percentile	
	PCS	MCS	PCS	MCS	PCS	MCS
**Analyzed cohort** [*N* = 5508]	**39.8** | 9.9 | 0.90	47.7 | 10.6 | 0.89	10.3 | 66.4	14.0 | 73.7	32.0 | 39.7 | 47.7	39.9 | 48.8 | 56.4
**Diagnosis**						
Angina [*N* = 1836]	**38.5** | 9.5 | 0.89	47.2 | 10.7 | 0.89	11.9 | 66.4	17.2 | 73.1	31.3 | 38.0 | 45.5	39.5 | 48.4 | 56.2
MI [*N* = 2086]	43.5 | 9.3 | 0.90	47.9 | 10.3 | 0.89	12.6 | 63.8	14.0 | 71.9	36.6 | 44.2 | 50.9	40.6 | 49.2 | 56.4
Heart failure [*N* = 1586]	**36.5** | 9.6 | 0.89	47.9 | 10.7 | 0.88	10.3 | 60.3	17.4 | 73.7	29.3 | 35.3 | 43.7	39.5 | 49.0 | 56.7
**Gender and diagnosis**						
*Female* [*N* = 1312]	**37.0** | 9.9 | 0.90	45.9 | 10.9 | 0.88	13.7 | 63.1	17.2 | 73.7	29.5 | 36.0 | 44.4	37.5 | 46.7 | 54.7
Angina [*N* = 494]	**35.8** | 9.5	45.7 | 11.2	14.7 | 63.1	17.2 | 68.3	29.2 | 34.3 | 42.5	37.4 | 46.3 | 54.8
MI [*N* = 480]	40.8 | 9.7	45.6 | 10.5	15.0 | 62.1	21.0 | 67.1	33.1 | 41.1 | 48.5	37.1 | 46.7 | 54.6
Heart failure [*N* = 338]	**33.4** | 9.0	46.6 | 11.1	13.7 | 57.5	17.6 | 73.7	27.1 | 31.9 | 39.3	38.0 | 47.7 | 55.2
*Male* [*N* = 4196]	40.6 | 9.7 | 0.90	48.2 | 10.4 | 0.89	10.3 | 66.4	14.0 | 73.1	33.1 | 40.7 | 48.5	40.7 | 49.6 | 56.8
Angina [*N* = 1342]	**39.4** | 9.3	47.8 | 10.4	11.9 | 66.4	17.5 | 73.1	32.4 | 39.1 | 46.4	40.3 | 48.9 | 56.6
MI [*N* = 1606]	44.2 | 9.0	48.6 | 10.2	12.6 | 63.8	14.0 | 71.9	37.8 | 45.2 | 51.6	41.8 | 50.4 | 56.8
Heart failure [*N* = 1248]	**37.3** | 9.5	48.3 |10.6	10.3 | 60.3	17.4 | 70.8	30.1 | 36.3 | 44.6	39.8 | 49.5 | 57.0
**Age and diagnosis**						
*<51 years* [*N* = 866]	42.3 | 9.7 | 0.91	45.9 | 10.9 | 0.90	15.6 | 62.1	16.6 | 68.9	35.1 | 42.4 | 50.5	37.8 | 47.3 | 55.1
Angina [*N* = 222]	**39.5** | 9.8	44.1 | 10.9	18.2 | 60.9	19.3 | 64.7	31.6 | 38.9 | 47.3	36.6 | 45.5 | 53.2
MI [*N* = 465]	45.2 | 8.9	47.0 | 10.8	15.6 | 62.1	16.6 | 66.9	38.6 | 46.8 | 52.6	39.7 | 48.2 | 55.9
Heart failure [*N* = 179]	**38.4** | 9.4	45.2 | 11.0	19.9 | 58.0	22.2 | 68.9	30.3 | 38.4 | 45.7	36.0 | 45.8 | 53.8
*51–60 years* [*N* = 1656]	40.1 | 9.6 | 0.90	46.4 | 10.7 | 0.90	11.9 | 66.4	14.0 | 71.5	32.6 | 40.2 | 47.6	38.5 | 47.0 | 55.6
Angina [*N* = 579]	**38.2** | 9.3	45.2 | 10.9	11.9 | 66.4	17.2 | 67.3	30.9 | 37.5 | 45.2	36.6 | 45.1 | 54.8
MI [*N* = 705]	43.6 | 8.9	47.3 | 10.3	15.8 | 63.8	14.0 | 71.5	33.2 | 40.0 | 47.0	39.6 | 48.0 | 56.1
Heart failure [*N* = 372]	**36.8** | 9.4	46.5 | 10.8	14.0 | 59.3	17.4 | 70.8	28.2 | 32.6 | 38.1	38.5 | 47.0 | 55.3
*61–70 years* [*N* = 1676]	**39.7** | 9.9 | 0.90	48.7 | 10.2 | 0.88	11.5 | 62.4	19.1 | 71.9	32.0 | 39.3 | 47.9	41.2 | 50.2 | 57.0
Angina [*N* = 608]	**39.0** | 9.5	48.8 | 10.2	13.5 | 61.5	19.1 | 69.8	32.0 | 38.5 | 46.2	41.9 | 50.4 | 57.0
MI [*N* = 562]	43.3 | 9.5	48.8 | 10.0	14.1 | 62.4	21.0 | 71.9	35.8 | 44.2 | 51.1	41.7 | 50.5 | 57.0
Heart failure [*N* = 506]	**36.4** | 9.7	48.5 | 10.4	11.5 | 57.9	23.8 | 69.0	29.4 | 35.6 | 44.2	39.8 | 49.8 | 57.0
*>70 years* [*N* = 1310]	**37.8** | 9.8 | 0.89	49.2 | 10.3 | 0.87	10.3 | 60.3	18.4 | 73.7	30.1 | 37.3 | 45.3	41.9 | 50.4 | 57.8
Angina [*N* = 427]	**37.6** | 9.5	49.4 | 10.1	16.3 | 58.1	21.8 | 73.1	30.3 | 37.3 | 44.6	42.4 | 50.9 | 57.8
MI [*N* = 354]	41.2 | 9.8	48.8 | 10.0	12.6 | 60.2	18.4 | 66.5	33.3 | 41.9 | 49.1	41.9 | 50.0 | 57.5
Heart failure [*N* = 529]	**35.6** | 9.5	48.5 | 10.4	10.3 | 60.3	20.6 | 73.7	28.6 | 34.3 | 42.5	41.3 | 50.3 | 58.0
**Age, gender and diagnosis**						
*<51 years and female* [*N* = 195]	41.0 | 10.3	45.0 | 10.9	15.6 | 62.1	19.6 | 63.8	32.9 | 40.6 | 49.4	37.8 | 47.6 | 53.4
Angina [*N* = 62]	**38.9** | 10.2	43.6 | 11.9	18.1 | 60.9	19.6 | 63.1	30.6 | 37.4 | 46.8	36.6 | 46.4 | 53.2
MI [*N* = 96]	43.6 | 10.0	45.0 | 10.3	15.6 | 62.1	21.3 | 62.7	37.3 | 44.5 | 52.1	40.7 | 46.8 | 53.5
Heart failure [*N* = 37]	**37.5** | 9.3	47.6 | 10.4	20.1 | 55.0	24.6 | 63.8	29.7 | 36.4 | 46.1	35.2 | 50.4 | 53.7
*<51 years and male* [*N* = 671]	42.7 | 9.5	46.1 | 10.9	16.2 | 62.1	16.6 | 68.9	35.7 | 42.8 | 50.6	37.8 | 47.3 | 55.5
Angina [*N* = 160]	**39.7** | 9.7	44.3 | 10.5	21.1 | 58.8	20.1 | 64.7	31.9 | 39.1 | 47.6	36.5 | 44.6 | 52.9
MI [*N* = 369]	45.6 | 8.5	47.5 | 10.9	16.2 | 62.1	16.6 | 66.9	39.3 | 46.9 | 52.8	40.7 | 50.0 | 56.2
Heart failure [*N* = 142]	**38.6** | 9.4	45.2 | 11.1	19.9 | 58.0	22.2 | 68.9	30.5 | 39.3 | 45.7	35.2 | 44.8 | 53.8
*51–60 years and female* [*N* = 342]	**37.2** | 9.6	43.6 | 11.2	14.7 | 63.1	17.2 | 70.1	30.1 | 35.2 | 44.3	34.8 | 42.8 | 52.3
Angina [*N* = 139]	**35.8** | 9.5	42.7 | 11.1	14.7 | 63.1	17.2 | 65.8	30.6 | 37.4 | 46.8	37.8 | 46.3 | 55.5
MI [*N* = 135]	40.6 | 9.3	45.0 | 10.6	15.8 | 59.6	22.6 | 65.0	37.3 | 44.5 | 52.1	40.3 | 49.0 | 56.4
Heart failure [*N* = 68]	**33.5** | 8.0	42.9 | 12.4	19.3 | 56.3	17.6 | 70.1	29.7 | 36.4 | 46.1	39.4 | 47.9 | 55.4
*51–60 years and male* [*N* = 1314]	40.9 | 9.5	47.1 | 10.4	11.9 | 66.4	14.0 | 71.5	33.7 | 41.1 | 48.1	39.4 | 47.7 | 55.9
Angina [*N* = 440]	**38.9** | 9.2	46.0 | 10.7	11.9 | 66.4	17.5 | 67.3	32.0 | 38.7 | 45.5	37.8 | 46.3 | 55.5
MI [*N* = 570]	44.3 | 8.7	47.8 | 10.2	19.4 | 63.8	14.0 | 71.5	38.3 | 44.9 | 50.9	40.3 | 49.0 | 56.4
Heart failure [*N* = 304]	**37.5** | 9.5	47.3 | 10.3	14.0 | 59.3	17.4 | 70.8	30.2 | 36.3 | 54.8	39.4 | 47.9 | 55.4
*61–70 years and female* [*N* = 421]	**36.6** | 9.8	46.0 | 11.0	13.7 | 57.8	20.3 | 67.1	29.3 | 35.2 | 44.2	37.4 | 46.4 | 55.2
Angina [*N* = 174]	**36.3** | 9.3	46.7 | 11.2	16.7 | 57.8	20.3 | 66.0	29.4 | 34.5 | 43.2	38.3 | 47.0 | 56.9
MI [*N* = 133]	40.0 | 9.6	44.9 | 10.8	15.0 | 57.4	21.0 | 67.1	31.2 | 40.9 | 47.6	36.7 | 45.7 | 53.7
Heart failure [*N* = 114]	**33.2** | 9.5	46.3 | 10.7	13.7 | 57.5	24.0 | 66.0	26.4 | 31.2 | 40.2	37.8 | 47.2 | 54.4
*61–70 years and male* [*N* = 1255]	40.7 | 9.8	49.6 | 9.7	11.5 | 62.4	19.1 | 71.9	33.4 | 40.5 | 48.9	43.3 | 51.2 | 57.4
Angina [*N* = 434]	40.1 | 9.3	49.6 | 9.6	13.5 | 61.5	19.1 | 69.8	33.9 | 39.5 | 47.4	44.0 | 51.2 | 57.0
MI [*N* = 429]	44.3 | 9.3	50.0 | 9.5	14.1 | 62.4	21.6 | 71.9	37.5 | 46.0 | 51.8	44.0 | 52.0 | 57.7
Heart failure [*N* = 392]	**37.4** | 9.5	49.1 | 10.2	11.5 | 57.9	23.8 | 69.0	30.4 | 36.6 | 44.8	40.2 | 50.4 | 57.5
*>70 years and female* [*N* = 354]	**35.2** | 9.6	48.4 | 10.2	15.7 | 60.2	20.4 | 73.7	27.7 | 33.7 | 42.3	41.6 | 49.2 | 56.7
Angina [*N* = 119]	**33.6** | 8.9	48.9 | 10.1	16.3 | 56.9	23.4 | 68.3	26.4 | 33.0 | 39.8	42.2 | 49.6 | 56.9
MI [*N* = 116]	**39.8** | 9.7	47.6 | 10.1	20.5 | 60.2	23.0 | 66.5	31.6 | 40.9 | 47.9	40.1 | 47.9 | 56.7
Heart failure [*N* = 119]	**32.2** | 8.5	48.6 | 10.4	15.7 | 55.4	20.4 | 73.7	26.6 | 31.1 | 37.6	41.6 | 49.7 | 57.1
*>70 years and male* [*N* = 956]	**38.7** | 9.8	49.5 | 10.3	10.3 | 60.3	18.4 | 73.1	31.4 | 38.4 | 46.2	42.0 | 50.8 | 57.9
Angina [*N* = 308]	**39.1** | 9.2	49.7 | 10.2	18.8 | 58.1	21.8 | 73.1	32.0 | 38.9 | 45.8	42.6 | 51.3 | 57.8
MI [*N* = 238]	41.9 | 9.8	49.4 | 10.0	12.6 | 59.9	18.4 | 66.2	34.2 | 42.5 | 50.0	42.7 | 50.9 | 58.0
Heart failure [*N* = 410]	**36.6** | 9.6	49.5 | 10.6	10.3 | 60.3	23.6 | 68.5	29.7 | 35.2 | 43.6	41.1 | 50.1 | 58.4

*MI* myocardial infarction, *N* number of patients

#### Mean PCS and MCS scores

The mean PCS score in the cohort was 39.8 ± 9.9, more than one SD below the standardized M of 50, and the mean MCS score of 47.7 ± 10.6 was within the normal range of 40–60.

#### Diagnosis

A main effect of diagnosis on mean PCS scores was found (ANOVA; *F*(2, 5505) = 274, *p* < 0.001, *η*_*p*_^2^ = 0.091). Post hoc analyses using BC indicated that mean PCS scores of patients with MI were significantly higher than in patients with either angina (*p* < 0.001, *d* = 0.53) or ischemic heart failure (*p* < 0.001; *d* = 0.74) who also had a significantly lower mean PCS score compared to patients with angina (*p* < 0.001, *d* = 0.21). All MCS scores were within the normal range and did not differ statistically (ANOVA; *F*(2, 5505) = 2.5, *p* = 0.083, *η*_*p*_^2^ = 0.001).

#### Gender

An independent-samples *t* test indicated that mean PCS scores were significantly lower for women (37.0 ± 9.9) than for men (40.6 ± 9.7; *t*(5506) = 11.7, *p* < 0.001, *d* = 0.37). Although mean MCS scores were in the normal range for both genders, female patients reported lower scores (45.9 ± 10.9) than male patients (48.2 ± 10.4; *t*(2101) = 6.9, *p* < 0.001, *d* = 0.22).

#### Age

A main effect of age on mean PCS scores was found (ANOVA; *F*(3, 5504) = 38.4, *p* < 0.001, *η*_*p*_^2^ = 0.020). According to post hoc analyses, patients <51 years reported significantly higher mean PCS scores compared to all other age groups (BC, all *p* < 0.001; vs. 51–60 years *d* = 0.22; vs. 61–70 years *d* = 0.27; vs. > 70 years *d* = 0.46). Patients >70 years had the lowest mean PCS scores (BC, all *p* < 0.001; vs. 51–60 years *d* = 0.24; vs. 61–70 years *d* = 0.19). A main effect of age was also found on mean MCS scores (ANOVA; *F*(3, 5504) = 31.6, *p* < 0.001, *η*_*p*_^2^ = 0.017) with patients <51 and 51–60 years reporting significantly lower MCS scores than both older aged groups (BC, all *p* < 0.001; <51 vs. 61–70 years *d* = 0.27 and vs. > 70 years *d* = 0.32; 51–60 vs. 61–70 years *d* = 0.22 and vs. >70 years: *d* = 0.27).

#### Interaction effects

Sub-analyses investigating interaction effects of diagnosis × gender, diagnosis × age, gender × age, diagnosis × gender × age showed no significant results for either PCS or MCS scale, except for gender × age on PCS (MANOVA; *F*(3,5500) = 4.19, *p* = 0.006, *η*_*p*_^2^ = 0.002) and diagnosis × age on MCS (MANOVA; *F*(6,5496) = 2.71, *p* = 0.012, *η*_*p*_^2^ = 0.003). However, the effect sizes for both are negligible.

### SF-36 component summary measures: region and country within region (Table 3)

All M ± SD, minimum, maximum, 25th, 50th and 75th percentile PCS/MCS scores and Cronbach’s Alpha for each region and country are given in Table 3. All means were tested for possible influences of the different data collecting sites, and all differences were less than the minimal important difference.

**Table 3:** Physical component summary (PCS) and mental component summary (MCS) mean scores, standard deviations and Cronbach’s Alpha grouped by region and countries (mean PCS or MCS scores >1 standard deviation below the standardized mean of 50 are bold)

	Mean | standard deviation | α		Min | max score		25th | 50th | 75th percentile	
	PCS	MCS	PCS	MCS	PCS	MCS
**Eastern Europe** [*N* = 1160]	**35.9** | 8.5 | 0.87	44.7 | 10.0 | 0.88	14.0 | 61.4	17.6 | 70.8	29.5 | 34.4 | 41.7	37.0 | 45.1 | 52.5
Hungary [*N* = 263]	**35.5** | 8.6 | 0.89	45.4 | 11.8 | 0.90	14.0 | 57.7	19.7 | 70.8	29.4 | 34.7 | 41.4	35.8 | 45.9 | 55.4
Poland [*N* = 314]	**38.1** | 10.1 | 0.88	46.3 | 9.9 | 0.88	18.8 | 60.5	21.1 | 65.6	30.0 | 36.9 | 45.7	39.5 | 47.4 | 54.0
Russia [*N* = 304]	**34.1** | 6.9 | 0.86	43.3 | 9.6 | 0.89	18.3 | 59.3	20.6 | 63.2	29.0 | 33.1 | 38.2	35.9 | 42.9 | 50.7
Ukraine [*N* = 279]	**35.6** | 7.6 | 0.86	43.8 | 8.5 | 0.87	18.2 | 61.4	17.6 | 63.9	29.9 | 34.3 | 41.9	37.5 | 43.7 | 50.5
**English speaking** [*N* = 1231]	**39.7** | 10.9 | 0.92	49.8 | 10.1 | 0.87	13.0 | 63.8	17.9 | 73.7	30.5 | 40.0 | 48.8	42.3 | 51.4 | 58.2
Australia [*N* = 220]	**38.1** | 11.2 | 0.90	48.1 | 10.8 | 0.87	16.2 | 63.8	22.6 | 66.2	28.1 | 35.5 | 48.7	39.6 | 49.4 | 57.8
Canada [*N* = 328]	42.4 | 10.5 | 0.91	51.0 | 9.4 | 0.87	13.0 | 61.6	21.6 | 67.2	35.9 | 44.0 | 50.6	45.3 | 53.5 | 58.5
Ireland/UK [*N* = 266]	**38.4** | 11.0 | 0.92	50.6 | 9.8 | 0.86	14.1 | 62.4	17.9 | 66.9	29.5 | 38.5 | 47.4	44.8 | 52.2 | 58.2
USA [*N* = 417]	**39.2** | 10.6 | 0.92	49.3 | 10.3 | 0.88	14.9 | 61.6	19.6 | 73.7	30.3 | 38.7 | 48.0	41.5 | 50.9 | 57.9
**Scandinavia** [*N* = 856]	40.5 | 10.1 | 0.91	49.6 | 10.0 | 0.89	10.9 | 60.2	21.6 | 73.1	32.6 | 40.8 | 48.8	42.4 | 50.7 | 57.7
Denmark [*N* = 277]	42.2 | 9.7 | 0.92	50.3 | 9.8 | 0.90	20.2 | 58.1	21.6 | 73.1	34.3 | 43.0 | 50.8	43.7 | 51.9 | 57.9
Norway [*N* = 294]	**38.4** | 10.2 | 0.90	49.6 | 9.9 | 0.88	10.9 | 59.3	23.8 | 70.5	30.5 | 39.2 | 45.7	42.3 | 50.7 | 57.6
Sweden [*N* = 285]	40.9 | 9.9 | 0.91	49.0 | 10.3 | 0.88	12.6 | 60.2	24.2 | 70.5	33.0 | 40.9 | 48.9	41.2 | 50.3 | 57.8
**Southern Europe** [*N* = 793]	40.9 | 9.2 | 0.88	47.9 | 10.5 | 0.88	10.3 | 61.7	17.4 | 68.9	33.9 | 41.1 | 48.6	40.9 | 49.4 | 56.7
Italy [*N* = 258]	42.0 | 9.2 | 0.87	46.9 | 9.8 | 0.86	19.2 | 59.6	24.2 | 68.9	35.0 | 42.8 | 49.8	39.4 | 47.9 | 54.4
Portugal [*N* = 277]	40.2 | 8.8 | 0.88	45.1 | 11.0 | 0.90	19.5 | 61.7	17.4 | 65.2	33.4 | 39.7 | 47.3	37.6 | 45.4 | 54.8
Spain [*N* = 258]	40.6 | 10.0 | 0.90	51.9 | 9.5 | 0.88	10.3 | 60.3	20.1 | 66.7	32.7 | 41.1 | 48.5	46.4 | 54.0 | 59.1
*Cuba* [*N* = 159]	**38.4** | 8.6 | 0.85	49.2 | 11.2 | 0.85	19.1 | 55.8	18.4 | 71.5	33.0 | 38.0 | 45.3	43.5 | 49.8 | 57.5
**Western Europe** [*N* = 1309]	42.4 | 9.3 | 0.90	46.7 | 11.0 | 0.91	11.9 | 66.4	14.0 | 70.5	35.4 | 42.7 | 49.9	38.6 | 47.8 | 56.0
Aust/Ger/Swiz [*N* = 329]	42.8 | 9.1 | 0.89	46.6 | 10.8 | 0.92	17.9 | 62.1	21.0 | 65.8	35.6 | 43.1 | 50.2	38.7 | 48.0 | 56.3
Belgium [*N* = 304]	42.8 | 9.7 | 0.90	50.1 | 10.0 | 0.89	19.3 | 63.1	16.6 | 68.1	35.4 | 43.3 | 50.8	43.7 | 52.1 | 58.1
France [*N* = 345]	41.2 | 8.0 | 0.87	41.3 |10.6 | 0.89	22.2 | 59.6	14.0 | 67.2	35.5 | 41.0 | 47.3	33.0 | 41.1 | 49.6
Netherlands [*N* = 331]	42.8 | 10.2 | 0.92	49.4 | 10.2 | 0.91	11.9 | 66.4	22.0 | 70.5	34.6 | 44.0 | 50.9	43.2 | 51.5 | 57.3

*Aust/Ger/Swiz* Austria/Germany/Switzerland, *N* number of patients, *UK* United Kingdom, *USA* United States of America

#### PCS scores

By region, the mean PCS score was more than one SD below the standardized *M* = 50 in Eastern Europe and the English-speaking region. The mean PCS score in EE was 35.9 ± 8.5, with each country in this region scoring more than one SD below *M* = 50. In the ES region, the mean PCS score was 39.7 ± 10.9, with only Canada within the normal range. In Sc, the mean PCS score was 40.5 ± 10.1; only Norway scored more than one SD below *M* = 50. In SE and WE, the mean PCS scores (40.9 ± 9.2; 42.4 ± 9.3) in all countries were within the normal range. In Cuba, the mean PCS score was 38.4 ± 8.6, significantly different from Spain (40.6 ± 10.0; *t*(372) = 2.41, *p* = 0.016, *d* = 0.37).

A main effect of the region (ANOVA; *F*(4, 5503) = 73.6, *p* < 0.001, *η*_*p*_^2^ = 0.051) and the country (ANOVA; *F*(17, 5490) = 23.8, *p* < 0.001, *η*_*p*_^2^ = 0.069) was found on mean PCS scores. Post hoc analyses indicated that patients in EE had significantly lower mean PCS scores than in each other region (BC, all *p* < 0.001; vs. ES, *d* = 0.39; vs. Sc, *d* = 0.49; vs. SE, *d* = 0.56; vs. WE, *d* = 0.73) and patients in WE had the highest mean PCS score when compared to each other region (BC, all *p* < 0.01; vs. ES, *d* = 0.27; vs. Sc, *d* = 0.20; vs. SE, *d* = 0.16). Except Hungary and Ukraine, the lowest PCS score in Russia was significantly lower than in all other countries (*p* < 0.001); Austria/Germany/Switzerland’s, the Netherlands’ and Belgium’s PCS scores were significantly higher than those reported in Russia, Hungary, Ukraine, Australia, Poland, Ireland/UK, Norway and the USA (all *p* < 0.001).

#### MCS scores

By region, each mean MCS score was within one SD± the standardized *M* = 50, ranging from 44.7 ± 10.0 in EE to 49.8 ± 10.1 in the ES region. A main effect of region (ANOVA; *F*(4, 5503) = 47.5, *p* < 0.001, *η*_*p*_^2^ = 0.033) and country (ANOVA; *F*(17, 5490) = 26.2, *p* < 0.001, *η*_*p*_^2^ = 0.075) was found on mean MCS scores. Post hoc analyses indicated that patients in EE had significantly lower mean MCS scores than in each other region although they were all within the normal range (BC, all *p* < 0.001; vs. ES *d* = 0.50; vs. Sc *d* = 0.49; vs. SE *d* = 0.31; vs. WE *d* = 0.19). Patients in the ES region had higher mean MCS scores than SE and WE patients (BC, all *p* < 0.01; vs. SE *d* = 0.18; vs. WE *d* = 0.29), and so did patients from Sc (BC, all *p* < 0.02; vs. SE *d* = 0.17; vs. WE *d* = 0.28). France had the significantly lowest mean MCS score compared to all other countries (*p* < 0.001), except for Russia and Ukraine. Patients in Spain, Canada and Ireland/UK had significantly the highest mean MCS scores when compared to patients from France, Russia, Ukraine, Portugal, Hungary, Austria/Germany/Switzerland and Italy (all *p* < 0.001).

### SF-36 scales: age, gender, diagnosis, region and country (Table 4)

The raw values of the eight scales give more detailed information than PCS and MCS values and are presented in detail in Table 4. As further country-specific reference data would be helpful, supplementary material for each country presenting reference values by diagnosis, age and gender separately are available [online resource 1].

**Table 4:** SF-36 scales (raw values 0–100) grouped by diagnosis, gender, age and region including countries

	Mean | standard deviation							
	Scales in the physical component summary measure (PCS)				Scales in the mental component summary measure (MCS)			
	PFI	ROLPH	PAIN	GHP	VITAL	SOCIAL	ROLEM	MHI
**Diagnosis**								
Angina [*N* = 1836]	62.3 | 23.8	39.2 | 40.9	56.8 | 24.6	50.1 | 20.1	51.5 | 20.2	73.1 | 23.7	57.3 | 42.9	67.5 | 19.1
MI [*N* = 2086]	73.4 | 22.3	43.5 | 41.9	71.7 | 25.0	58.6 | 20.2	57.9 | 20.6	76.9 | 23.1	61.0 | 42.3	71.0 | 18.8
Heart failure [*N* = 1586]	52.9 | 24.6	32.9 | 40.1	64.1 | 26.1	46.3 | 19.2	49.7 | 20.5	71.5 | 24.2	56.3 | 43.4	68.7 | 19.6
**Gender**								
Female [*N* = 1312]	53.7 | 25.1	32.9 | 40.0	59.1 | 26.4	48.6 | 19.9	47.9 | 20.4	70.5 | 24.6	50.6 | 43.4	64.7 | 19.9
Male [*N* = 4196]	67.0 | 24.0	41.0 | 41.5	66.2 | 25.6	53.4 | 25.1	55.1 | 20.5	75.2 | 23.3	60.8 | 42.4	70.6 | 18.7
**Age**								
<51 years [*N* = 866]	72.2 | 23.4	41.0 | 41.0	67.2 | 25.9	52.5 | 20.8	54.0 | 20.7	73.7 | 23.8	58.7 | 43.0	66.0 | 19.7
51–60 years [*N* = 1656]	66.2 | 23.7	37.6 | 40.6	62.4 | 25.9	51.6 | 20.7	52.9 | 20.6	72.7 | 23.7	55.2 | 42.6	67.1 | 19.3
61–70 years [*N* = 1676]	63.6 | 24.9	40.7 | 42.2	65.0 | 25.6	52.7 | 20.4	54.0 | 21.0	75.6 | 23.6	61.7 | 42.5	70.6 | 18.5
>70 years [*N* = 1310]	55.5 | 25.0	37.5 | 41.1	64.9 | 26.3	52.2 | 20.5	52.8 | 20.6	74.1 | 23.8	58.1 | 43.3	72.0 | 18.8
**Eastern Europe**								
Hungary [*N* = 263]	57.9 | 22.8	23.7 | 33.6	53.3 | 26.2	39.4 | 17.5	52.0 | 22.2	70.3 | 25.4	37.5 | 40.9	66.8 | 21.4
Poland [*N* = 314]	57.8 | 24.8	39.0 | 44.5	57.2 | 28.1	49.0 | 16.7	53.3 | 18.2	69.7 | 25.6	62.2 | 43.5	60.6 | 17.8
Russia [*N* = 304]	50.3 | 21.5	22.3 | 33.9	49.2 | 19.4	41.0 | 12.0	45.8 | 17.6	61.4 | 22.6	46.1 | 42.3	57.7 | 15.9
Ukraine [*N* = 279]	53.6 | 21.6	28.9 | 37.8	52.5 | 21.8	40.5 | 13.4	48.7 | 18.1	63.9 | 20.2	41.5 | 40.3	62.2 | 15.4
**English speaking**								
Australia [*N* = 220]	57.0 | 28.7	36.6 | 42.6	64.3 | 26.6	53.4 | 22.4	48.5 | 21.9	71.4 | 25.8	55.2 | 44.9	72.7 | 17.9
Canada [*N* = 328]	71.9 | 23.3	46.8 | 40.7	72.3 | 22.8	59.8 | 21.7	56.1 | 20.3	80.5 | 21.7	70.6 | 39.2	76.9 | 15.4
Ireland/UK [*N* = 266]	59.0 | 26.8	40.5 | 40.1	68.2 | 26.5	53.2 | 23.1	51.6 | 21.8	74.8 | 23.9	68.4 | 39.5	74.6 | 17.6
USA [*N* = 417]	58.8 | 27.4	45.0 | 42.7	64.4 | 25.0	54.7 | 22.2	52.8 | 20.4	76.0 | 23.8	59.4 | 44.1	73.1 | 17.9
**Scandinavia**								
Denmark [*N* = 277]	69.3 | 25.6	45.4 | 41.8	72.4 | 23.4	57.2 | 20.9	56.7 | 23.3	85.2 | 20.4	61.2 | 38.7	75.4 | 18.5
Norway [*N* = 294]	67.2 | 24.7	26.7 | 37.3	61.6 | 27.7	56.7 | 22.4	49.6 | 20.3	77.0 | 22.5	57.0 | 42.5	76.5 | 16.7
Sweden [*N* = 285]	64.8 | 23.0	42.6 | 40.0	67.8 | 25.5	56.9 | 21.0	54.2 | 21.0	78,7 | 21.9	56.9 | 41.9	73.8 | 17.7
**Southern Europe**								
Italy [*N* = 258]	66.3 | 24.4	44.4 | 41.7	70.3 | 26.5	53.3 | 20.0	57.7 | 19.1	72.9 | 22.6	57.9 | 42.7	67.7 | 17.1
Portugal [*N* = 277]	59.1 | 24.2	46.1 | 41.9	65.7 | 26.0	46.4 | 17.9	51.4 | 20.9	74.1 | 23.4	53.1 | 41.4	62.4 | 22.1
Spain [*N* = 258]	69.6 | 20.8	51.6 | 43.0	67.5 | 25.4	51.2 | 21.6	56.8 | 22.7	81.9 | 21.7	81.9 | 35.7	72.7 | 18.4
*Cuba* [*N* = 159]	66.9 | 20.3	23.3 | 35.7	63.3 | 26.3	52.8 | 16.5	61.1 | 23.7	70.7 | 29.0	81.1 | 34.3	61.0 | 22.6
**Western Europe**								
Aut/Ger/Swiz [*N* = 329]	70.3 | 23.4	44.6 | 42.1	68.8 | 28.1	56.7 | 17.9	52.8 | 20.7	77.8 | 23.3	59.1 | 43.6	67.6 | 19.4
Belgium [*N* = 304]	70.5 | 24.5	48.5 | 42.0	71.7 | 22.5	56.8 | 20.3	62.9 | 18.3	79.5 | 20.4	65.5 | 40.6	73.2 | 17.7
France [*N* = 345]	71.4 | 20.9	27.6 | 36.8	58.1 | 22.5	52.7 | 18.9	46.7 | 17.5	65.6 | 22.6	37.5 | 41.8	62.4 | 19.4
Netherlands [*N* = 331]	68.8 | 25.3	48.3 | 42.0	76.2 | 23.4	56.9 | 21.8	58.9 | 19.4	74.8 | 21.3	68.3 | 41.1	73.3 | 18.0

Scales in the PCS: PFI, Physical Functioning; ROLPH, Role-Physical; PAIN, Bodily Pain; GHP, General Health

Scales in the MCS: VITAL, Vitality; SOCIAL, Social Functioning; ROLEM, Role-Emotional; MHI, Mental Health

*Aust/Ger/Swiz* Austria/Germany/Switzerland, *N* number of patients, *UK* United Kingdom, *USA* United States of America

## Discussion

The key to the interpretation of HRQL as an outcome is having reference values as these are useful for users of an instrument who wish to place their results in an appropriate context by comparing their scores to a reference group [6]. Without reference values, it is difficult for the user, whether clinicians, researchers, or policy makers, to assess the meaning of the comparative or relative scores. This is the first dedicated study representing generic HRQL SF-36 international IHD reference values allowing comparisons across angina, MI and ischemic heart failure as well as across 22 countries in one publication. The pattern of all HRQL differences observed in this report is similar to findings of other studies using the SF-36 [15, 16] or SF-12 [20]. The SF-36 scores reported here by 5508 patients substantiate findings from other studies (e.g., EuroAspire) [20], therefore contributing to the establishment of relationships between HRQL and variables such as diagnosis, age, gender or region.

The mean PCS score in the analyzed cohort with IHD was below the normal range of 40–60, whereas the mean MCS score was within this range. Diagnosis exerted an influence on physical health with patients with MI always reporting the highest mean PCS scores. However, patients with angina and ischemic heart failure were more than one SD below the standardized PCS mean score. On the other hand, cardiac diagnosis had no effect on the mean MCS scores which were all within the normal range. These results indicate that angina and especially ischemic heart failure have a greater impact on physical health than MI. This might be due to their different subsequent physical limitations and chronicity. Furthermore, cardiac diseases apparently have a stronger influence on physical than on mental health. Females reported worse physical and mental health than males. This maybe indicates more subjective perceived physical and mental burden in women. Worse physical health was also reported by older patients (especially in those >70 years), whereas younger patients (especially in those <51 years) had lower mean MCS scores in comparison. These results may lead to the assumption that younger patients are fitter and therefore can handle physical strain due to a cardiac disease better than older patients. In contrast, older patients perceive less mental stress because of more coping strategies available or the feeling of “normality” when they are confronted with a cardiac disease at a greater age. Finally, patients from EE had significantly lower physical and mental health scores than patients from all other regions. These PCS and MCS results, with patients from EE are reporting worse HRQL than patients from the other regions, are consistent with the well-recognized East–West health divide [13, 24].

The well-known East–West Europe health divide between Eastern Europe, i.e., the formerly communist countries of Central and Eastern Europe and the former Soviet Union [24, 25], and Western Europe reflects major differences in health policy, access to and quality of health care, health care funding, and certain health risks and their impact on health outcomes such as life expectancy, morbidity and mortality. The East–West Europe health divide was confirmed for HRQL as a patient-reported outcome measure in this report. The combined effects of economic growth, improved health care and successful health policies (e.g., tobacco and alcohol control, food policy, road traffic safety) across Western Europe have resulted in a higher life expectancy, lower mortality and morbidity and healthier populations [20, 24]. In contrast, economic and political problems in many Eastern European countries have frequently led to a failure to implement effective health policies with concomitant lower life expectancies, higher mortality and morbidity and less healthy populations [26]. However, inequities in health, health care and health care policies also exist within and between neighboring countries in Western Europe [24, 27, 28] and remarkable HRQL differences between neighboring countries have been noted in this analysis indicating further challenges to health, well-being and health care in Western Europe [20].

The generic SF-36 is useful when comparing various populations with a healthy cohort. The SF-36 PCS and MCS measures, with a standardized mean of 50 ± 10, are derived from the general “healthy” US population [8]. There are SF-36 norms by IHD diagnosis, i.e., angina, MI and heart failure, in the German [7] and US [8] population substantiating the findings in this study of highest scores in patients with MI, followed by patients with angina, and the lowest scores in patients with heart failure. Furthermore, in the German [7] and US [8] general populations, females reported worse PCS and MCS scores than males and younger patients reported better PCS and worse MCS scores older patients. This analysis revealed similar results within an IHD population. Moreover, there are also PCS and MCS norms broken down into their components for the eight SF-36 scales by diagnosis (heart disease in total, MI and heart failure), age and gender in the US population [8]. The reference values generated in the present study demonstrated that all PCS scores, but none of the MCS scores, were more than one SD lower than the general “healthy” US population norms in patients with angina and heart failure. However, the SF-36 does not quantify symptom burden or functional limitations specific to IHD and is less sensitive to clinical change, either over time or after a therapeutic intervention, and its clinical interpretation is more difficult than with a disease-specific instrument [6] such as either the MacNew [29] or the HeartQoL [21, 22]. Furthermore, when the SF-36 scales are summated and transformed to the higher-order PCS and MCS measures, the formula always includes the means, the SDs and the regression coefficients from the general American population (“US weights”). This needs to be considered when interpreting these or other SF-36 data in countries other than the USA as, when interpreting SF-36 data across different groups (e.g., gender, ethnicity, language), problems may occur because people belonging to different groups may have a different probability of giving a certain response on a questionnaire [30]. By generating new IHD-specific reference values in the future, where a standardized mean of 50 ± 10 would represent the “average IHD-patient,” these specific SF-36 reference data could be used for comparing scores within an IHD population more precisely.

Therefore, analyses of measurement invariance or differential item functioning can be conducted to provide an indication of unexpected behavior of items on a test and giving information on which items may be revised, e.g., exclusion, rephrasing, new translation. There is apparently a valid assumption based on the vast literature that the SF-36 at least does measure the same construct across different cultures (IQOLA-Project) [31–33]. Moreover, the Bjorner et al. DIF-study [26] showed that the use of homogenous reduced scales instead of the full SF-36 scales (containing items with DIF) did not change conclusions about the existence of a cross-national difference, at least not between the general populations in the USA and Denmark. These data can lead to the assumption that the SF-36 is also robust when locating items with DIF regarding the “higher-ordered” results (scales, PCS/MCS) in other populations. But as the study of Bjorner et al. [26] is unique with results suggesting that cross-language DIF may be a frequent problem in questionnaire translations, some consequences occur. Therefore, validations of translated instruments are needed every time a questionnaire is presented in a new language and interpretations of cross-national comparison data need to be used with caution since the interpretation of questions and the use of response categories may vary between countries.

## Limitations

The data are based on convenience samples. This needs to be considered when referring to these results. For example, only patients with ischemic heart failure (left ventricular dysfunction <40 % and IHD) were included, whereas other heart failure diagnoses were excluded (e.g., preserved ejection fraction or diastolic heart failure, right heart failure). Furthermore disease severity, also influencing physical and mental health, varied across regions in this sample. The highest proportion of patients with severe angina (CCS III + IV) was found in the ES region (36 %) and the lowest in Sc (20 %). The highest proportion of patients with severe ischemic heart failure (NYHA III + IV) was found in EE (47 %) and the lowest in Sc (28 %).

The 876 excluded participants were more likely to be female, older, less likely to be married and higher educated. They reported worse health status (single SF-36 question “In general, would you say your health is…excellent/very good/good/fair/poor?”), came more often from ES countries, Sc and SE and less often from EE and WE, were less likely to have hypercholesterolemia but more likely to be diabetic and physical inactive. Excluding these patients because of incomplete data or being outliers could have influenced the presented reference values.

## Conclusions

In 5508 patients with angina, MI and ischemic heart failure, the diagnosis exerted a significant influence on the perception of physical health with the highest mean SF-36 PCS scores reported by patients with MI and the poorest scores reported by patients with heart failure. Worse physical health was also reported by females, older patients (especially those >70 years) and patients from EE. The cardiac diagnosis had no effect on the mean MCS scores which were all within the normal range; however, females, younger patients (especially those <51 years) and EE patients reported the lowest mean MCS scores. Clinicians, researchers, health professionals and health-policy makers can use these SF-36 reference values for patients with IHD as an indication of how an individual patient, or a group of patients, compares to patients of the same sex, age and diagnosis as well as to patients with IHD in a specific country. Important health challenges that need to be addressed remain in both Eastern and Western Europe countries concerning unresolved issues in health-policy-making and rising health inequalities between and within countries.

## Electronic supplementary material

Below is the link to the electronic supplementary material.

Supplementary material 1 (PDF 374 kb)
