# Supplementary material for: International SF-36 reference values in patients with ischemic heart disease
Source: Qual Life Res. 2016 Jun 18;25(11):2787–98. doi: 10.1007/s11136-016-1316-4 (PMC5065592; doi:10.1007/s11136-016-1316-4)
Supplement: Supplementary file 1 — Supplementary material 1 (PDF 374 kb) [file 11136_2016_1316_MOESM1_ESM.pdf]

Supplementary Material

1. Australia: Physical Component Summary (PCS) and Mental Component Summary (MCS) measure grouped by diagnosis, gender and age (cells with bold scores indicate >1 standard deviation below the standardized mean of 50)

	**Mean | Standard deviation**		**Min | Max scores**		**25th | 50th | 75th percentile**	
**Australia** [N=220]	**PCS**	**MCS**	**PCS**	**MCS**	**PCS**	**MCS**
**Diagnosis**						
Angina [N=58]	**35.9** | 10.1	46.2 | 10.4	19.3 | 58.8	26.5 | 64.6	27.5 | 33.2 | 44.6	37.3 | 47.8 | 55.2
MI [N=87]	41.1 | 11.9	48.5 | 10.9	16.2 | 63.8	22.6 | 66.2	29.8 | 40.9 | 52.8	40.0 | 47.6 | 58.9
Heart failure [N=75]	**36.3** | 10.4	49.2 | 11.0	20.2 | 57.9	23.6 | 65.7	27.4 | 34.5 | 44.7	39.8 | 51.6 | 57.8
**Gender**						
Female [N=44]	**34.5** | 10.4	44.8 | 10.7	19.9 | 57.0	26.5 | 65.7	26.2 | 31.1 | 43.6	35.4 | 44.5 | 51.4
Male [N=176]	**39.0** | 11.2	49.0 | 10.7	16.2 | 63.8	22.6 | 66.2	29.4 | 37.2 | 49.5	40.0 | 50.4 | 58.3
**Age**						
< 51 years [N=22]	44.3 | 11.2	47.3 | 11.6	16.2 | 58.8	26.5 | 63.0	38.6 | 48.6 | 52.3	39.4 | 46.8 | 59.2
51-60 years [N=47]	**39.6** | 11.6	46.4 | 10.9	21.3 | 63.8	26.5 | 64.2	29.6 | 38.1 | 51.8	37.1 | 47.9 | 56.6
61-70 years [N=68]	**37.5** | 11.1	48.6 | 11.0	22.1 | 59.0	22.6 | 65.7	27.6 | 34.8 | 48.1	40.3 | 49.8 | 57.5
> 70 years [N=83]	**36.0** | 10.4	48.9 | 10.5	19.3 | 58.9	23.6 | 66.2	27.7 | 34.2 | 43.1	39.7 | 50.2 | 57.9

International SF-36 reference values in patients with ischemic heart disease; *Quality of Life Research*; **A.**, **Huber**[a,b], N., Oldridge, & S., Höfer. (2016).
[a] Department of Medical Psychology, Medical University of Innsbruck, Speckbacherstraße 23/III, 6020 Innsbruck, Austria
[b] Institute of Psychology, Leopold Franzens University Innsbruck, Innrain 52f, Bruno-Sander-Haus, 6020 Innsbruck, Austria
E-Mail: alexandra.huber@i-med.ac.at

2. Austria, Germany & Switzerland: Physical Component Summary (PCS) and Mental Component Summary (MCS) measure grouped by diagnosis, gender and age (cells with bold scores indicate >1 standard deviation below the standardized mean of 50)

	**Mean | Standard deviation**		**Min | Max scores**		**25th | 50th | 75th percentile**	
**AUT / GER / CH** [N=329]	**PCS**	**MCS**	**PCS**	**MCS**	**PCS**	**MCS**
**Diagnosis**						
Angina [N=105]	41.3 | 9.0	46.3 | 10.7	20.3 | 60.4	21.7 | 64.9	34.9 | 40.9 | 49.0	38.7 | 47.9 | 55.3
MI [N=131]	46.4 | 8.0	47.4 | 10.7	26.5 | 62.1	21.8 | 65.0	41.9 | 47.3 | 53.0	40.9 | 48.8 | 57.2
Heart failure [N=93]	**39.5** | 9.1	46.0 | 11.0	17.9 | 56.7	21.0 | 65.8	33.0 | 39.5 | 46.6	36.9 | 47.3 | 55.4
**Gender**						
Female [N=45]	41.6 | 9.6	43.6 | 10.2	22.2 | 60.4	22.6 | 65.0	33.1 | 41.7 | 48.6	36.5 | 42.4 | 51.8
Male [N=284]	43.0 | 9.1	47.1 | 10.8	17.9 | 62.1	21.0 | 65.8	36.1 | 48.6 | 56.4	39.9 | 48.6 | 56.4
**Age**						
< 51 years [N=89]	44.4 | 9.3	44.0 | 10.8	21.7 | 62.1	21.8 | 62.2	36.8 | 44.3 | 51.8	34.9 | 45.0 | 52.3
51-60 years [N=126]	41.6 | 9.3	45.8 | 11.2	17.9 | 61.7	21.0 | 65.8	35.1 | 41.2 | 48.5	37.6 | 46.3 | 55.8
61-70 years [N=81]	44.1 | 8.5	49.9 | 9.2	21.4 | 58.7	27.6 | 63.1	37.1 | 45.2 | 50.7	43.0 | 52.2 | 57.5
> 70 years [N=33]	40.2 | 8.8	49.2 | 10.5	25.6 | 55.4	29.8 | 64.0	32.5 | 40.0 | 47.3	40.0 | 50.8 | 58.2

3. Belgium: Physical Component Summary (PCS) and Mental Component Summary (MCS) measure grouped by diagnosis, gender and age (cells with bold scores indicate >1 standard deviation below the standardized mean of 50)

	**Mean | Standard deviation**		**Min | Max scores**		**25th | 50th | 75th percentile**	
**Belgium** [N=304]	**PCS**	**MCS**	**PCS**	**MCS**	**PCS**	**MCS**
**Diagnosis**						
Angina [N=91]	42.9 | 10.0	48.7 | 11.0	20.1 | 63.1	17.2 | 65.6	35.4 | 43.9 | 51.9	40.3 | 51.9 | 57.6
MI [N=126]	46.1 | 8.2	50.7 | 9.6	21.4 | 60.8	16.6 | 64.6	40.5 | 47.6 | 52.3	44.7 | 52.9 | 58.3
Heart failure [N=87]	**37.8** | 9.5	50.5 | 9.4	19.3 | 57.2	29.4 | 68.1	31.5 | 38.8 | 46.0	43.9 | 50.9 | 58.3
**Gender**						
Female [N=56]	41.5 | 10.7	49.0 | 9.9	20.1 | 63.1	17.2 | 64.1	32.0 | 42.8 | 49.8	42.2 | 50.0 | 57.7
Male [N=248]	43.0 | 9.5	50.3 | 10.0	19.3 | 62.6	16.6 | 68.0	35.4 | 43.5 | 51.0	43.8 | 52.4 | 58.3
**Age**						
< 51 years [N=42]	43.7 | 10.3	47.0 | 10.6	20.1 | 60.8	16.6 | 63.3	34.9 | 43.1 | 53.3	39.4 | 47.8 | 55.7
51-60 years [N=92]	44.5 | 9.1	49.2 | 10.9	20.1 | 63.1	17.2 | 66.4	39.6 | 44.2 | 50.2	41.9 | 52.0 | 58.0
61-70 years [N=93]	44.6 | 8.5	50.9 | 9.2	19.3 | 57.6	27.2 | 64.6	38.7 | 45.9 | 51.7	45.0 | 53.5 | 58.5
> 70 years [N=77]	**37.9** | 10.1	51.6 | 8.9	19.9 | 58.2	29.4 | 68.1	31.1 | 35.7 | 45.6	45.4 | 53.7 | 59.0

4. Canada: Physical Component Summary (PCS) and Mental Component Summary (MCS) measure grouped by diagnosis, gender and age (cells with bold scores indicate >1 standard deviation below the standardized mean of 50)

	**Mean | Standard deviation**		**Min | Max scores**		**25th | 50th | 75th percentile**	
**Canada** [N=328]	**PCS**	**MCS**	**PCS**	**MCS**	**PCS**	**MCS**
**Diagnosis**						
Angina [N=95]	43.1 | 10.2	51.5 | 9.7	13.5 | 61.5	51.5 | 9.7	37.0 | 45.0 | 50.1	47.0 | 53.4 | 58.8
MI [N=139]	46.3 | 9.0	50.1 | 9.1	15.6 | 61.6	21.6 | 62.5	41.1 | 48.1 | 53.0	46.3 | 53.1 | 57.9
Heart failure [N=94]	**36.1** | 10.0	51.1 | 9.6	13.0 | 55.5	28.1 | 67.2	29.4 | 36.5 | 43.3	43.0 | 53.7 | 59.2
**Gender**						
Female [N=67]	40.4 | 10.5	50.5 | 9.7	15.6 | 58.9	27.0 | 64.6	32.6 | 41.8 | 48.5	43.3 | 52.2 | 57.9
Male [N=261]	43.0 | 10.5	51.1 | 9.4	13.0 | 61.6	21.6 | 67.2	36.7 | 44.8 | 50.9	46.0 | 53.6 | 58.6
**Age**						
< 51 years [N=48]	45.1 | 9.9	48.9 | 9.7	15.6 | 61.6	27.2 | 61.8	41.1 | 46.7 | 51.2	44.6 | 50.6 | 56.6
51-60 years [N=89]	44.7 | 9.6	49.5 | 9.3	16.9 | 61.2	27.0 | 64.7	38.1 | 45.6 | 53.5	41.9 | 50.9 | 56.9
61-70 years [N=103]	41.0 | 11.3	51.3 | 10.1	13.5 | 61.5	21.6 | 66.8	33.6 | 43.2 | 50.9	44.3 | 54.7 | 59.3
> 70 years [N=88]	40.3 | 10.3	53.2 | 8.2	13.0 | 58.9	28.9 | 67.2	34.2 | 41.9 | 48.4	47.8 | 55.5 | 59.2

5. Cuba: Physical Component Summary (PCS) and Mental Component Summary (MCS) measure grouped by diagnosis, gender and age (cells with bold scores indicate >1 standard deviation below the standardized mean of 50)

	**Mean | Standard deviation**		**Min | Max scores**		**25th | 50th | 75th percentile**	
**Cuba** [N=159]	**PCS**	**MCS**	**PCS**	**MCS**	**PCS**	**MCS**
**Diagnosis**						
Angina [N=57]	**39.4** | 9.1	48.4 | 9.9	19.7 | 55.8	22.1 | 65.1	32.7 | 39.5 | 46.6	43.2 | 49.1 | 55.3
MI [N=74]	40.0 | 7.2	50.4 | 12.1	19.4 | 54.6	18.4 | 71.5	34.2 | 39.3 | 45.9	44.9 | 51.4 | 59.6
Heart failure [N=28]	**32.2** | 8.5	47.9 | 11.4	19.1 | 51.1	24.1 | 66.0	25.5 | 32.2 | 37.6	40.0 | 49.7 | 56.5
**Gender**						
Female [N=32]	**35.9** | 8.6	42.8 | 11.3	19.1 | 54.6	22.1 | 64.6	29.5 | 35.1 | 42.3	31.6 | 44.3 | 50.6
Male [N=127]	**39.1** | 8.5	50.9 | 10.6	19.4 | 55.8	18.4 | 71.5	33.6 | 39.2 | 46.0	45.0 | 50.9 | 58.5
**Age**						
< 51 years [N=33]	41.9 | 7.5	47.8 | 11.9	26.8 | 54.6	19.1 | 66.0	37.5 | 42.0 | 48.0	44.7 | 49.2 | 55.8
51-60 years [N=42]	**39.0** | 10.0	50.1 | 12.5	19.4 | 55.8	19.3 | 71.5	32.8 | 39.8 | 46.5	42.3 | 52.2 | 60.0
61-70 years [N=61]	**36.1** | 7.7	51.3 | 9.0	19.1 | 53.7	28.3 | 66.2	31.1 | 35.7 | 42.1	45.5 | 50.2 | 59.5
> 70 years [N=23]	**38.5** | 8.1	44.4 | 11.6	19.7 | 51.5	18.4 | 61.6	33.5 | 37.7 | 44.1	34.1 | 46.3 | 54.7

6. Denmark: Physical Component Summary (PCS) and Mental Component Summary (MCS) measure grouped by diagnosis, gender and age (cells with bold scores indicate >1 standard deviation below the standardized mean of 50)

	**Mean | Standard deviation**		**Min | Max scores**		**25th | 50th | 75th percentile**	
**Denmark** [N=277]	**PCS**	**MCS**	**PCS**	**MCS**	**PCS**	**MCS**
**Diagnosis**						
Angina [N=111]	41.2 | 8.7	49.6 | 9.8	21.5 | 56.3	22.6 | 73.1	34.9 | 41.0 | 48.8	43.2 | 51.6 | 57.5
MI [N=90]	45.8 | 10.1	50.9 | 8.6	20.2 | 58.1	30.3 | 65.9	44.2 | 52.9 | 58.1	44.2 | 52.9 | 58.1
Heart failure [N=76]	**39.5** | 9.4	50.5 | 11.0	22.3 | 57.5	21.6 | 67.0	31.7 | 37.2 | 48.2	43.9 | 50.6 | 60.4
**Gender**						
Female [N=67]	**38.8** | 9.9	47.1 | 11.2	21.0 | 57.4	21.6 | 66.1	30.3 | 38.9 | 45.8	39.8 | 45.9 | 56.8
Male [N=210]	43.3 | 9.4	51.3 | 9.1	20.2 | 58.1	27.4 | 73.1	35.5 | 45.0 | 51.0	45.2 | 53.4 | 58.0
**Age**						
< 51 years [N=36]	45.0 | 10.4	47.1 | 10.3	24.8 | 58.1	22.6 | 65.6	36.0 | 49.0 | 54.4	39.2 | 45.2 | 56.6
51-60 years [N=66]	42.9 | 8.5	48.8 | 9.9	24.6 | 57.2	21.6 | 65.3	35.6 | 44.1 | 50.6	43.3 | 50.5 | 57.0
61-70 years [N=89]	43.9 | 9.8	52.3 | 9.3	21.7 | 58.0	28.9 | 64.6	36.9 | 46.4 | 52.0	45.4 | 54.9 | 60.3
> 70 years [N=86]	**38.8** | 9.3	50.8 | 9.7	20.2 | 56.6	27.4 | 73.1	31.1 | 38.3 | 45.9	43.7 | 51.0 | 57.4

7. France: Physical Component Summary (PCS) and Mental Component Summary (MCS) measure grouped by diagnosis, gender and age (cells with bold scores indicate >1 standard deviation below the standardized mean of 50)

	**Mean | Standard deviation**		**Min | Max scores**		**25th | 50th | 75th percentile**	
**France** [N=345]	**PCS**	**MCS**	**PCS**	**MCS**	**PCS**	**MCS**
**Diagnosis**						
Angina [N=97]	40.1 | 7.3	40.4 | 10.4	25.6 | 56.7	20.3 | 66.0	34.3 | 40.0 | 44.6	33.2 | 40.4 | 47.6
MI [N=148]	43.4 | 7.8	40.8 | 10.6	24.5 | 59.6	14.0 | 66.5	38.0 | 43.3 | 49.8	31.8 | 40.8 | 48.1
Heart failure [N=100]	**38.9** | 8.0	42.8 | 10.7	22.2 | 54.4	23.0 | 67.2	33.0 | 38.8 | 45.1	33.5 | 42.1 | 51.8
**Gender**						
Female [N=62]	40.4 | 8.8	41.2 | 11.6	22.2 | 59.6	20.3 | 66.5	33.4 | 39.2 | 47.3	32.6 | 40.2 | 50.7
Male [N=283]	41.3 | 7.8	41.3 | 10.3	23.5 | 57.8	14.0 | 67.2	35.8 | 41.5 | 47.3	33.1 | 41.5 | 49.5
**Age**						
< 51 years [N=80]	42.6 | 7.6	40.2 | 10.7	26.6 | 55.3	19.9 | 62.7	36.5 | 42.0 | 49.3	30.7 | 39.6 | 47.9
51-60 years [N=112]	41.1 | 8.3	40.6 | 10.9	22.2 | 59.6	14.0 | 62.7	35.5 | 41.9 | 46.4	32.6 | 39.5 | 48.4
61-70 years [N=99]	41.0 | 8.4	43.1 | 9.7	23.5 | 55.1	20.3 | 65.7	35.0 | 40.2 | 48.6	35.0 | 43.5 | 51.3
> 70 years [N=54]	**39.5** | 6.9	40.8 | 11.0	27.4 | 56.7	22.5 | 67.2	34.9 | 39.4 | 43.8	33.0 | 40.2 | 46.8

8. Hungary: Physical Component Summary (PCS) and Mental Component Summary (MCS) measure grouped by diagnosis, gender and age (cells with bold scores indicate >1 standard deviation below the standardized mean of 50)

	**Mean | Standard deviation**		**Min | Max scores**		**25th | 50th | 75th percentile**	
**Hungary** [N=263]	**PCS**	**MCS**	**PCS**	**MCS**	**PCS**	**MCS**
**Diagnosis**						
Angina [N=89]	**33.4** | 7.7	43.2 | 11.3	14.7 | 50.0	20.0 | 63.6	28.2 | 33.0 | 39.1	34.4 | 43.3 | 53.0
MI [N=92]	**39.6** | 8.6	46.2 | 12.4	18.1 | 57.7	19.7 | 66.9	31.4 | 40.3 | 45.3	37.0 | 47.8 | 57.6
Heart failure [N=82]	**33.2** | 7.9	46.9 | 11.6	14.0 | 50.2	23.8 | 70.8	26.9 | 32.9 | 39.7	38.2 | 48.2 | 55.5
**Gender**						
Female [N=120]	**32.5** | 7.9	43.2 | 11.8	14.7 | 54.9	20.0 | 64.8	26.8 | 30.5 | 38.4	33.6 | 42.2 | 52.8
Male [N=143]	**38.0** | 8.4	47.2 | 11.6	14.0 | 57.7	19.7 | 70.8	32.1 | 38.2 | 44.6	38.3 | 48.0 | 57.4
**Age**						
< 51 years [N=26]	**38.9** | 9.9	47.9 | 12.4	18.1 | 55.4	22.1 | 66.9	31.9 | 38.2 | 47.5	41.2 | 50.1 | 56.4
51-60 years [N=98]	**37.1** | 9.0	45.2 | 12.2	14.0 | 57.7	19.7 | 70.8	30.4 | 37.2 | 44.6	34.7 | 46.3 | 56.4
61-70 years [N=86]	**35.2** | 7.0	44.6 | 11.9	20.9 | 52.7	21.0 | 64.8	30.4 | 33.7 | 40.1	35.7 | 43.7 | 55.4
> 70 years [N=53]	**31.4** | 8.0	45.8 | 10.9	19.8 | 55.9	24.1 | 63.6	25.6 | 29.5 | 38.5	36.5 | 47.9 | 55.0

9. Ireland & UK: Physical Component Summary (PCS) and Mental Component Summary (MCS) measure grouped by diagnosis, gender and age (cells with bold scores indicate >1 standard deviation below the standardized mean of 50)

	**Mean | Standard deviation**		**Min | Max scores**		**25th | 50th | 75th percentile**	
**Ireland & UK** [N=266]	**PCS**	**MCS**	**PCS**	**MCS**	**PCS**	**MCS**
**Diagnosis**						
Angina [N=91]	**37.8** | 10.3	49.6 | 10.8	18.8 | 58.4	19.1 | 66.0	29.9 | 36.8 | 44.9	43.7 | 52.2 | 57.0
MI [N=102]	42.3 | 10.5	49.8 | 9.2	14.1 | 62.4	17.9 | 62.1	34.9 | 45.6 | 49.7	44.5 | 50.8 | 57.7
Heart failure [N=73]	**33.7** | 10.7	53.1 | 9.2	14.8 | 55.8	30.8 | 66.9	25.4 | 32.1 | 41.8	46.9 | 53.9 | 61.2
**Gender**						
Female [N=72]	**37.9** | 10.0	50.6 | 10.5	15.7 | 54.8	24.1 | 66.9	30.3 | 37.2 | 46.2	41.5 | 52.2 | 59.3
Male [N=194]	**38.6** | 11.4	50.6 | 9.6	14.1 | 62.4	17.9 | 66.8	28.9 | 38.9 | 47.5	45.1 | 52.1 | 57.9
**Age**						
< 51 years [N=36]	40.4 | 10.9	46.1 | 11.7	20.4 | 58.4	17.9 | 61.6	32.0 | 39.2 | 49.6	37.8 | 48.7 | 56.2
51-60 years [N=71]	**38.4** | 10.6	49.3 | 9.3	20.0 | 57.8	23.6 | 65.8	29.9 | 40.9 | 48.7	43.7 | 50.3 | 56.7
61-70 years [N=92]	**38.6** | 11.5	50.8 | 10.0	14.0 | 62.4	19.1 | 65.5	28.9 | 39.3 | 48.7	45.2 | 52.6 | 59.1
> 70 years [N=67]	**36.5** | 10.9	54.1 | 7.9	15.7 | 56.2	34.0 | 66.9	28.2 | 36.8 | 46.2	48.8 | 55.1 | 60.1

10. Italy: Physical Component Summary (PCS) and Mental Component Summary (MCS) measure grouped by diagnosis, gender and age (cells with bold scores indicate >1 standard deviation below the standardized mean of 50)

	**Mean | Standard deviation**		**Min | Max scores**		**25th | 50th | 75th percentile**	
**Italy** [N=258]	**PCS**	**MCS**	**PCS**	**MCS**	**PCS**	**MCS**
**Diagnosis**						
Angina [N=85]	**39.7** | 9.4	48.3 | 9.6	19.2 | 59.6	24.2 | 66.3	33.4 | 40.1 | 46.9	42.3 | 49.5 | 54.9
MI [N=96]	46.1 | 8.1	46.7 | 9.1	24.7 | 59.6	25.6 | 61.7	41.3 | 47.6 | 52.2	40.3 | 47.3 | 54.1
Heart failure [N=77]	**39.4** | 8.5	45.5 | 10.8	19.8 | 57.0	24.4 | 68.9	32.9 | 39.8 | 45.9	37.1 | 45.3 | 54.9
**Gender**						
Female [N=57]	**38.6** | 10.6	44.1 | 10.0	19.6 | 54.2	24.2 | 63.1	28.9 | 40.9 | 48.0	35.8 | 45.3 | 51.6
Male [N=201]	42.9 | 8.5	47.6 | 9.6	19.2 | 59.6	24.4 | 68.9	36.3 | 43.0 | 50.2	40.5 | 48.8 | 55.2
**Age**						
< 51 years [N=28]	47.6 | 7.1	48.4 | 10.9	30.1 | 59.1	26.4 | 68.9	42.2 | 49.4 | 53.6	38.7 | 51.2 | 56.3
51-60 years [N=63]	41.4 | 8.7	47.3 | 9.9	19.8 | 59.6	24.2 | 65.5	35.9 | 41.2 | 49.2	40.3 | 47.2 | 55.5
61-70 years [N=93]	41.6 | 9.2	46.7 | 9.2	19.2 | 59.6	25.6 | 64.5	34.4 | 42.6 | 49.2	39.2 | 48.4 | 53.4
> 70 years [N=74]	40.9 | 9.6	46.1 | 10.1	19.6 | 56.3	24.4 | 66.3	33.6 | 42.8 | 48.8	40.1 | 45.6 | 54.2

11. Netherlands: Physical Component Summary (PCS) and Mental Component Summary (MCS) measure grouped by diagnosis, gender and age (cells with bold scores indicate >1 standard deviation below the standardized mean of 50)

	**Mean | Standard deviation**		**Min | Max scores**		**25th | 50th | 75th percentile**	
**Netherlands** [N=331]	**PCS**	**MCS**	**PCS**	**MCS**	**PCS**	**MCS**
**Diagnosis**						
Angina [N=93]	**37.4** | 9.5	48.3 | 10.1	11.9 | 66.4	22.6 | 67.8	29.9 | 37.3 | 44.7	43.0 | 49.8 | 56.0
MI [N=145]	48.5 | 7.4	49.4 | 10.6	29.1 | 62.1	22.0 | 70.5	44.8 | 49.6 | 54.2	41.4 | 51.9 | 57.9
Heart failure [N=93]	**39.4** | 10.1	50.7 | 9.7	17.2 | 58.9	26.3 | 67.2	31.9 | 40.1 | 46.0	45.9 | 52.4 | 58.2
**Gender**						
Female [N=68]	42.0 | 10.0	48.1 | 10.0	20.0 | 62.1	23.8 | 63.7	32.9 | 43.7 | 48.6	41.6 | 48.8 | 57.2
Male [N=263]	43.0 | 10.2	49.8 | 10.3	11.9 | 66.4	22.0 | 70.5	34.9 | 44.1 | 51.8	43.6 | 52.0 | 57.7
**Age**						
< 51 years [N=54]	44.5 | 9.7	44.8 | 11.9	19.9 | 62.1	22.0 | 65.3	38.2 | 45.5 | 52.2	34.7 | 46.3 | 55.8
51-60 years [N=110]	44.1 | 10.2	49.6 | 9.8	11.9 | 66.4	22.6 | 70.5	37.2 | 45.7 | 52.7	43.2 | 52.1 | 57.4
61-70 years [N=85]	42.5 | 9.6	50.5 | 9.9	18.9 | 61.5	24.7 | 67.8	33.3 | 44.6 | 50.7	45.9 | 51.7 | 57.6
> 70 years [N=82]	40.4 | 10.6	51.3 | 9.2	18.3 | 57.4	28.4 | 63.5	31.7 | 41.2 | 49.3	45.4 | 52.8 | 59.2

12. Norway: Physical Component Summary (PCS) and Mental Component Summary (MCS) measure grouped by diagnosis, gender and age (cells with bold scores indicate >1 standard deviation below the standardized mean of 50)

	**Mean | Standard deviation**		**Min | Max scores**		**25th | 50th | 75th percentile**	
**Norway** [N=294]	**PCS**	**MCS**	**PCS**	**MCS**	**PCS**	**MCS**
**Diagnosis**						
Angina [N=91]	**37.0** | 9.2	48.2 | 10.5	16.5 | 57.3	23.8 | 67.3	30.1 | 36.5 | 42.7	39.8 | 48.5 | 58.2
MI [N=115]	42.3 | 9.2	50.4 | 8.8	15.0 | 59.1	29.2 | 67.1	36.1 | 43.2 | 49.2	43.5 | 52.2 | 56.8
Heart failure [N=88]	**34.8** | 10.9	50.0 | 10.5	10.9 | 59.3	24.0 | 70.5	26.5 | 33.7 | 42.5	42.6 | 51.6 | 57.8
**Gender**						
Female [N=48]	**36.2** | 10.5	48.3 | 12.0	13.7 | 58.9	23.8 | 70.1	28.5 | 36.9 | 43.2	37.1 | 48.7 | 58.5
Male [N=246]	**38.9** | 10.1	49.8 | 9.4	10.9 | 59.3	24.0 | 70.5	31.4 | 39.4 | 46.4	43.1 | 50.8 | 57.6
**Age**						
< 51 years [N=28]	41.9 | 10.3	47.5 | 9.9	21.1 | 58.2	23.8 | 63.1	36.6 | 41.4 | 51.3	39.4 | 49.5 | 56.3
51-60 years [N=87]	**39.5** | 10.2	49.8 | 9.9	17.9 | 59.3	26.6 | 70.5	30.8 | 40.7 | 46.6	43.2 | 50.2 | 57.1
61-70 years [N=109]	**38.2** | 10.9	50.5 | 9.7	13.7 | 57.4	26.5 | 69.0	30.3 | 37.9 | 47.2	43.7 | 51.8 | 58.9
> 70 years [N=70]	**36.1** | 8.7	48.8 | 10.1	10.9 | 52.0	24.0 | 67.1	29.1 | 36.1 | 43.1	39.2 | 51.4 | 57.5

13. Poland: Physical Component Summary (PCS) and Mental Component Summary (MCS) measure grouped by diagnosis, gender and age (cells with bold scores indicate >1 standard deviation below the standardized mean of 50)

	**Mean | Standard deviation**		**Min | Max scores**		**25th | 50th | 75th percentile**	
**Poland** [N=314]	**PCS**	**MCS**	**PCS**	**MCS**	**PCS**	**MCS**
**Diagnosis**						
Angina [N=109]	**35.6** | 8.4	45.3 | 10.8	18.8 | 56.2	21.1 | 64.5	29.8 | 33.7 | 41.7	36.8 | 45.0 | 53.7
MI [N=112]	42.8 | 9.8	46.8 | 9.0	20.5 | 60.5	24.0 | 65.6	35.3 | 42.9 | 51.5	40.9 | 48.0 | 53.9
Heart failure [N=93]	**35.5** | 10.2	46.9 | 9.7	19.0 | 60.3	22.6 | 64.0	27.6 | 32.6 | 43.8	40.8 | 48.1 | 54.0
**Gender**						
Female [N=82]	**36.2** | 9.3	45.5 | 10.0	20.5 | 60.0	21.1 | 63.5	29.3 | 33.2 | 43.1	39.9 | 46.5 | 54.0
Male [N=232]	**38.8** | 10.3	46.6 | 9.8	18.8 | 60.5	22.6 | 65.6	30.1 | 38.3 | 46.9	39.3 | 47.9 | 54.0
**Age**						
< 51 years [N=55]	40.3 | 11.2	44.4 | 10.7	21.7 | 60.0	23.5 | 65.6	29.9 | 40.2 | 50.8	36.6 | 46.2 | 51.3
51-60 years [N=118]	**38.2** | 9.8	45.3 | 10.2	18.8 | 60.5	21.1 | 64.5	30.8 | 35.2 | 44.9	36.8 | 46.6 | 54.0
61-70 years [N=83]	**37.6** | 9.3	47.7 | 9.0	19.0 | 58.2	25.5 | 64.0	30.2 | 38.0 | 46.6	41.7 | 48.0 | 54.9
> 70 years [N=58]	**36.7** | 10.5	48.1 | 9.0	20.5 | 60.3	24.7 | 63.7	27.9 | 36.6 | 43.8	43.4 | 48.7 | 54.0

14. Portugal: Physical Component Summary (PCS) and Mental Component Summary (MCS) measure grouped by diagnosis, gender and age (cells with bold scores indicate >1 standard deviation below the standardized mean of 50)

	**Mean | Standard deviation**		**Min | Max scores**		**25th | 50th | 75th percentile**	
**Portugal** [N=277]	**PCS**	**MCS**	**PCS**	**MCS**	**PCS**	**MCS**
**Diagnosis**						
Angina [N=91]	**39.3** | 7.7	45.0 | 10.9	20.9 | 55.6	21.8 | 63.7	33.6 | 38.8 | 45.3	37.7 | 45.2 | 55.4
MI [N=107]	43.5 | 8.7	44.9 | 11.0	25.6 | 61.7	21.3 | 65.2	37.1 | 44.2 | 51.4	36.7 | 44.9 | 54.5
Heart failure [N=79]	**36.7** | 8.6	45.6 | 11.2	19.5 | 57.2	17.4 | 63.8	29.0 | 36.0 | 41.9	37.9 | 46.5 | 54.2
**Gender**						
Female [N=63]	**36.0** | 8.4	41.2 | 10.5	19.5 | 54.2	21.3 | 63.8	28.8 | 35.2 | 43.0	32.3 | 41.6 | 49.6
Male [N=214]	41.4 | 8.5	46.3 | 10.9	20.9 | 61.7	17.4 | 65.2	34.9 | 40.7 | 48.4	39.1 | 46.6 | 56.0
**Age**						
< 51 years [N=50]	**39.6** | 10.1	44.5 | 11.0	22.6 | 55.3	21.3 | 63.8	30.9 | 40.0 | 49.0	37.5 | 43.4 | 53.5
51-60 years [N=90]	40.6 | 8.0	43.8 | 10.9	25.6 | 61.7	17.4 | 64.5	34.5 | 39.7 | 46.5	36.3 | 43.9 | 52.5
61-70 years [N=80]	42.3 | 9.0	46.6 | 10.5	19.5 | 60.8	25.4 | 63.5	35.3 | 41.5 | 49.3	39.4 | 48.7 | 55.8
> 70 years [N=57]	**37.3** | 7.8	45.6 | 11.9	20.9 | 54.2	21.8 | 65.2	32.5 | 37.2 | 42.6	38.4 | 46.5 | 55.6

15. Russia: Physical Component Summary (PCS) and Mental Component Summary (MCS) measure grouped by diagnosis, gender and age (cells with bold scores indicate >1 standard deviation below the standardized mean of 50)

	**Mean | Standard deviation**		**Min | Max scores**		**25th | 50th | 75th percentile**	
**Russia** [N=304]	**PCS**	**MCS**	**PCS**	**MCS**	**PCS**	**MCS**
**Diagnosis**						
Angina [N=106]	**34.5** | 7.3	43.8 | 9.2	18.3 | 52.3	24.2 | 60.7	29.0 | 32.6 | 39.2	36.8 | 44.2 | 50.5
MI [N=100]	**36.1** | 6.6	43.2 | 9.9	22.6 | 59.3	23.7 | 60.4	31.2 | 35.6 | 39.6	33.7 | 43.7 | 52.4
Heart failure [N=98]	**31.8** | 6.1	42.8 | 9.9	22.9 | 51.4	20.6 | 63.2	27.8 | 30.2 | 35.7	35.9 | 42.0 | 48.9
**Gender**						
Female [N=84]	**31.6** | 6.2	40.1 | 8.5	18.3 | 52.3	20.6 | 58.9	28.5 | 30.3 | 34.2	34.8 | 39.2 | 46.4
Male [N=220]	**35.1** | 6.9	44.5 | 9.8	22.9 | 59.3	23.8 | 63.2	29.5 | 34.9 | 39.2	36.5 | 44.8 | 52.2
**Age**						
< 51 years [N=66]	**37.2** | 7.4	45.5 | 10.1	24.6 | 59.3	24.2 | 62.5	29.9 | 37.7 | 41.4	35.9 | 46.9 | 55.4
51-60 years [N=105]	**34.2** | 6.3	42.4 | 9.1	24.1 | 51.4	24.2 | 63.2	29.1 | 33.0 | 37.3	35.5 | 41.1 | 49.0
61-70 years [N=100]	**33.2** | 6.9	43.6 | 9.3	18.3 | 52.3	23.7 | 62.7	28.9 | 32.4 | 37.5	37.0 | 44.5 | 50.4
> 70 years [N=33]	**30.7** | 5.2	40.6 | 10.0	23.5 | 47.4	20.6 | 61.1	28.0 | 30.1 | 32.2	34.0 | 36.3 | 49.8

16. Spain: Physical Component Summary (PCS) and Mental Component Summary (MCS) measure grouped by diagnosis, gender and age (cells with bold scores indicate >1 standard deviation below the standardized mean of 50)

	Mean | Standard deviation		Min | Max scores		25th | 50th | 75th percentile	
**Spain** [N=258]	**PCS**	**MCS**	**PCS**	**MCS**	**PCS**	**MCS**
**Diagnosis**						
Angina [N=86]	**37.4** | 9.7	51.0 | 9.8	16.3 | 56.7	20.1 | 64.7	29.6 | 37.6 | 45.8	46.1 | 53.4 | 58.1
MI [N=105]	45.3 | 8.3	52.4 | 9.6	27.6 | 60.3	26.1 | 65.8	38.6 | 45.7 | 52.7	47.1 | 55.3 | 60.2
Heart failure [N=67]	**37.7** | 10.2	52.4 | 9.2	10.3 | 56.4	29.1 | 66.7	31.1 | 35.6 | 46.0	46.8 | 53.3 | 59.4
**Gender**						
Female [N=34]	**35.6** | 8.7	49.7 | 8.5	16.3 | 53.4	21.2 | 61.4	30.6 | 35.2 | 39.6	46.2 | 51.3 | 56.4
Male [N=224]	41.4 | 10.0	52.2 | 9.6	10.3 | 60.3	20.1 | 66.7	34.0 | 42.6 | 49.1	46.4 | 54.8 | 59.6
**Age**						
< 51 years [N=67]	45.2 | 9.0	53.1 | 8.6	24.4 | 58.9	20.1 | 65.8	38.6 | 45.5 | 53.1	48.5 | 54.7 | 59.5
51-60 years [N=65]	40.2 | 9.2	51.3 | 10.2	23.0 | 60.3	26.4 | 64.2	32.4 | 40.3 | 47.4	44.7 | 55.7 | 59.1
61-70 years [N=71]	**39.2** | 9.8	50.8 | 10.0	11.5 | 56.7	21.2 | 66.3	35.0 | 39.5 | 46.9	45.3 | 53.6 | 58.1
> 70 years [N=55]	**37.4** | 10.7	52.6 | 9.2	10.3 | 56.6	29.1 | 66.7	30.1 | 35.0 | 46.3	46.4 | 53.3 | 60.6

17. Sweden: Physical Component Summary (PCS) and Mental Component Summary (MCS) measure grouped by diagnosis, gender and age (cells with bold scores indicate >1 standard deviation below the standardized mean of 50)

	**Mean | Standard deviation**		**Min | Max scores**		**25th | 50th | 75th percentile**	
**Sweden** [N=285]	**PCS**	**MCS**	**PCS**	**MCS**	**PCS**	**MCS**
**Diagnosis**						
Angina [N=89]	40.2 | 9.3	49.9 | 11.1	20.4 | 57.3	26.3 | 70.5	32.7 | 38.8 | 47.9	41.1 | 51.7 | 59.3
MI [N=111]	44.1 | 9.1	47.9 | 10.1	12.6 | 60.2	24.2 | 62.7	39.4 | 44.4 | 51.0	38.8 | 50.5 | 56.3
Heart failure [N=85]	**37.3** | 10.4	49.5 | 9.7	19.5 | 55.2	27.1 | 66.0	28.5 | 34.2 | 47.3	43.8 | 49.7 | 57.7
**Gender**						
Female [N=71]	**38.6** | 10.0	46.4 | 11.0	15.8 | 60.2	24.2 | 66.0	30.0 | 40.0 | 45.3	36.8 | 49.3 | 56.3
Male [N=214]	41.6 | 9.8	49.8 | 9.9	12.6 | 59.9	26.3 | 70.5	34.3 | 41.4 | 49.9	43.4 | 50.8 | 58.6
**Age**						
< 51 years [N=18]	46.6 | 7.7	45.2 | 10.4	32.8 | 57.3	26.3 | 62.7	39.2 | 48.2 | 53.9	35.2 | 46.2 | 53.0
51-60 years [N=58]	41.0 | 9.0	46.8 | 10.6	15.8 | 59.0	26.5 | 65.1	34.5 | 41.2 | 48.0	36.7 | 46.3 | 55.9
61-70 years [N=91]	41.0 | 10.5	50.1 | 9.7	19.6 | 57.1	28.3 | 68.6	32.7 | 41.1 | 50.8	41.7 | 52.3 | 57.9
> 70 years [N=118]	**39.8** | 10.0	49.7 | 10.4	12.6 | 60.2	24.2 | 70.5	32.2 | 39.8 | 48.8	42.2 | 50.1 | 59.5

18. Ukraine: Physical Component Summary (PCS) and Mental Component Summary (MCS) measure grouped by diagnosis, gender and age (cells with bold scores indicate >1 standard deviation below the standardized mean of 50)

	**Mean | Standard deviation**		**Min | Max scores**		**25th | 50th | 75th percentile**	
**Ukraine** [N=279]	**PCS**	**MCS**	**PCS**	**MCS**	**PCS**	**MCS**
**Diagnosis**						
Angina [N=94]	**35.3** | 7.6	43.8 | 9.5	18.2 | 56.9	21.7 | 62.5	29.7 | 34.5 | 40.2	36.4 | 43.3 | 51.2
MI [N=97]	**36.7** | 7.5	44.9 | 7.2	23.2 | 61.4	29.9 | 61.7	30.9 | 35.1 | 42.9	39.0 | 45.1 | 50.3
Heart failure [N=88]	**34.7** | 7.8	42.6 | 8.6	21.8 | 50.5	17.6 | 63.9	28.4 | 33.4 | 42.0	36.7 | 40.6 | 49.7
**Gender**						
Female [N=87]	**34.1** | 8.0	42.1 | 8.8	18.2 | 61.4	17.6 | 62.1	27.5 | 32.6 | 38.7	35.3 | 42.8 | 49.1
Male [N=192]	**36.3** | 7.4	44.5 | 8.3	21.7 | 56.9	25.5 | 63.9	30.5 | 34.9 | 42.7	38.2 | 44.4 | 51.4
**Age**						
< 51 years [N=55]	**37.8** | 8.9	44.1 | 9.0	18.2 | 61.4	21.7 | 59.0	31.2 | 36.6 | 44.8	36.5 | 44.1 | 51.9
51-60 years [N=117]	**35.8** | 6.9	42.9 | 8.0	21.7 | 52.1	17.6 | 62.5	30.9 | 35.1 | 41.2	37.8 | 42.7 | 49.0
61-70 years [N=82]	**34.6** | 7.6	44.7 | 8.9	21.8 | 53.2	25.5 | 63.9	28.6 | 33.0 | 41.5	37.1 | 45.6 | 51.4
> 70 years [N=25]	**33.2** | 7.1	44.2 | 8.5	22.5 | 45.8	31.9 | 59.6	27.0 | 33.4 | 38.0	37.7 | 41.6 | 51.3

19. USA: Physical Component Summary (PCS) and Mental Component Summary (MCS) measure grouped by diagnosis, gender and age (cells with bold scores indicate >1 standard deviation below the standardized mean of 50)

	**Mean | Standard deviation**		**Min | Max scores**		**25th | 50th | 75th percentile**	
**USA** [N=417]	**PCS**	**MCS**	**PCS**	**MCS**	**PCS**	**MCS**
**Diagnosis**						
Angina [N=198]	**38.7** | 10.9	49.1 | 10.4	14.9 | 60.9	19.6 | 69.8	29.9 | 37.6 | 47.9	41.9 | 51.0 | 57.8
MI [N=109]	43.4 | 10.2	49.5 | 9.3	18.4 | 61.6	27.7 | 71.9	35.2 | 43.7 | 51.5	42.8 | 51.0 | 56.7
Heart failure [N=110]	**36.0** | 9.3	49.4 | 11.2	17.2 | 57.5	25.4 | 73.7	28.2 | 33.7 | 43.6	40.0 | 50.4 | 59.3
**Gender**						
Female [N=153]	**37.2** | 11.1	50.9 | 10.3	14.9 | 60.9	19.6 | 73.7	28.7 | 36.2 | 45.6	44.6 | 51.4 | 59.1
Male [N=264]	40.3 | 10.2	48.4 | 10.3	19.8 | 61.6	23.0 | 71.9	31.8 | 40.4 | 48.8	39.9 | 50.1 | 57.3
**Age**						
< 51 years [N=33]	40.5 | 10.6	44.5 | 11.1	18.4 | 60.9	19.6 | 61.5	33.6 | 39.5 | 49.2	35.0 | 45.4 | 55.4
51-60 years [N=100]	40.2 | 11.7	45.6 | 10.5	14.9 | 61.6	23.0 | 65.8	30.0 | 40.5 | 50.5	37.3 | 45.3 | 54.1
61-70 years [N=110]	40.4 | 10.5	51.6 | 10.0	16.7 | 59.3	25.4 | 71.9	31.7 | 41.1 | 49.5	45.7 | 53.9 | 59.1
> 70 years [N=174]	**37.6** | 9.9	50.9 | 9.5	17.2 | 57.9	29.0 | 73.7	29.7 | 37.1 | 44.9	43.3 | 51.9 | 58.9
